# A1 Noradrenergic Neurons Lesions Reduce Natriuresis and Hypertensive Responses to Hypernatremia in Rats

**DOI:** 10.1371/journal.pone.0073187

**Published:** 2013-09-10

**Authors:** Elaine Fernanda da Silva, André Henrique Freiria-Oliveira, Carlos Henrique Xavier Custódio, Paulo César Ghedini, Luiz Artur Mendes Bataus, Eduardo Colombari, Carlos Henrique de Castro, Diego Basile Colugnati, Daniel Alves Rosa, Sergio L. D. Cravo, Gustavo Rodrigues Pedrino

**Affiliations:** 1 Department of Physiological Sciences, Biological Sciences Institute, Federal University of Goiás,Goiânia, Goiás, Brazil; 2 Department of Physiology and Pathology, School of Dentistry, São Paulo State University, Araraquara, São Paulo, Brazil; 3 Department of Physiology, Federal University of São Paulo, São Paulo, São Paulo, Brazil; The University of Manchester, United Kingdom

## Abstract

Noradrenergic neurons in the caudal ventrolateral medulla (CVLM; A1 group) contribute to cardiovascular regulation. The present study assessed whether specific lesions in the A1 group altered the cardiovascular responses that were evoked by hypertonic saline (HS) infusion in non-anesthetized rats. Male Wistar rats (280–340 g) received nanoinjections of antidopamine-β-hydroxylase-saporin (A1 lesion, 0.105 ng.nL^−1^) or free saporin (sham, 0.021 ng.nL^−1^) into their CVLMs. Two weeks later, the rats were anesthetized (2% halothane in O_2_) and their femoral artery and vein were catheterized and led to exit subcutaneously between the scapulae. On the following day, the animals were submitted to HS infusion (3 M NaCl, 1.8 ml • kg^−1^, b.wt., for longer than 1 min). In the sham-group (n = 8), HS induced a sustained pressor response (ΔMAP: 35±3.6 and 11±1.8 mmHg, for 10 and 90 min after HS infusion, respectively; P<0.05 vs. baseline). Ten min after HS infusion, the pressor responses of the anti-DβH-saporin-treated rats (n = 11)were significantly smaller(ΔMAP: 18±1.4 mmHg; P<0.05 vs. baseline and vs. sham group), and at 90 min, their blood pressures reached baseline values (2±1.6 mmHg). Compared to the sham group, the natriuresis that was induced by HS was reduced in the lesioned group 60 min after the challenge (196±5.5 mM vs. 262±7.6 mM, respectively; P<0.05). In addition, A1-lesioned rats excreted only 47% of their sodium 90 min after HS infusion, while sham animals excreted 80% of their sodium. Immunohistochemical analysis confirmed a substantial destruction of the A1 cell group in the CVLM of rats that had been nanoinjected withanti-DβH-saporin. These results suggest that medullary noradrenergic A1 neurons are involved in the excitatory neural pathway that regulates hypertensive and natriuretic responses to acute changes in the composition of body fluid.

## Introduction

Sodium (Na^+^) cationsare the main ions for determining the osmolarity and volume of the extracellular compartment of complex organismsdue to their concentration and reduced permeability through plasmatic cell membranes. Variations in the concentration of these ions can cause an osmotic flux between intra- and extracellular compartments, which can affect all perfused tissues and may alter the volume, metabolism, and function of cells [1].

The central nervous system (CNS) detects variations in the volume, tonicity, and composition of the extracellular compartment through various peripheral and central receptors [2]–[5]. Once detected,these changes trigger a set of responses that reestablish physiological conditions. In mammals, a slight increase of 1–2% in the osmolarity of the plasma or a reduction of 8–10% in the volume of the extracellular compartment is sufficient to induce water intake [6], [7]. As for vegetative adjustments, an increase in the concentration of plasma sodium levels reducesthe sympathetic outflow to renal nerves [8]–[12] andstimulatesrenal vasodilation [3], [11], [13]–[17], thereby resulting in natriuresis [18]–[21] and diuresis [2], [22]. Humoral responses also contribute to the regulation of osmolarity by inhibiting the secretion of renin [23], atrial natriuretic peptides (ANP) [24]–[28], oxytocin (OT) [29]–[32], and vasopressin [32].

Experimental evidenceindicates that A1 noradrenergic neurons, which are localized in the caudal ventrolateral medulla (CVLM), play a key role in regulating cardiovascular homeostasis [12], [17], [33]. Studies on the gene expression of immediate activation genes showed that the A1 neuronal group was recruited by increasing osmolarity or plasmatic volume [34]–[37]. Moreover, important rolesfor the A1 noradrenergic neurons on renal vasodilatation and sympathoinhibition that is induced by hypernatremia have been shown [12], [17].

Neuroanatomical studies have demonstrated that the A1 noradrenergic neurons compose the central circuitry ofthe baroreflex and cardiopulmonary reflexes [38]–[40]. These neurons are reciprocally connected to hypothalamic structures that are involved in the control of cardiovascular, renal and neuroendocrine functions [41]–[48]. Thus, A1neuron projections to diencephalic regions represent an important link in the neural circuits that control body fluid homeostasis.

In the present study, we sought to evaluate the involvement ofthe A1 neuronal group in the cardiovascular and renal responses that were induced by acute hypernatremiain non-anesthetized rats. After damagingA1 noradrenergic neurons by nanoinjecting anti-dopamine beta hydroxylase (DβH)–saporin into the CVLM, we measured the cardiovascular responses and renal excretions that were induced by hypertonic saline (HS) infusion.

## Results

### Lesions of the medullary catecholaminergic neuronsinduced by nanoinjections of anti-DβH–saporin into the CVLM

**Figure 1:**
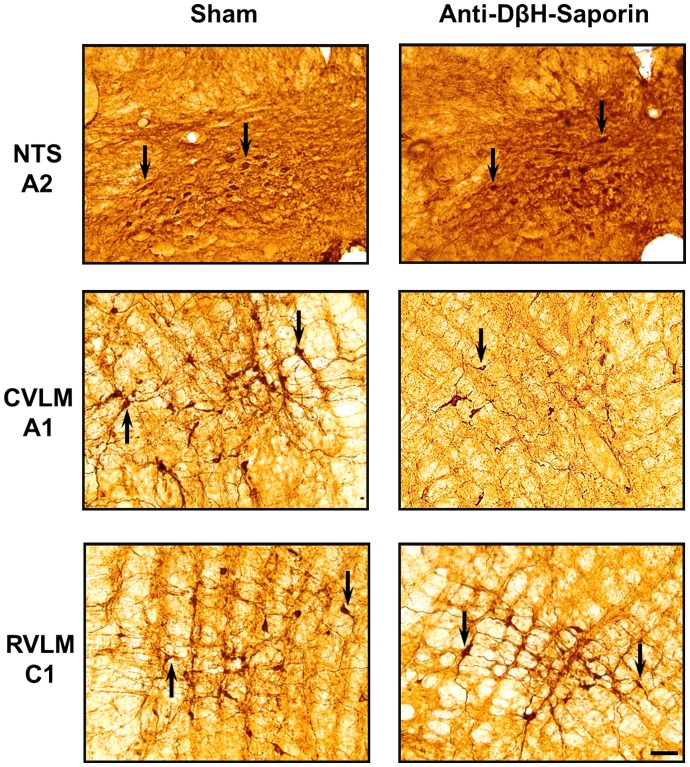
Medullary catecholaminergic neurons. Photomicrographs taken at 3 levels of the medulla showing TH-immunoreactive cells in the NTS (A2 noradrenergic neurons), CVLM (A1 noradrenergic neurons) and RVLM (C1 adrenergic neurons) in rats that were nanoinjected with unconjugated saporin (sham) or anti-DβH-saporin. Arrows indicate TH-positive cells, and the scale bar is equal to 50 µm.

**Figure 2:**
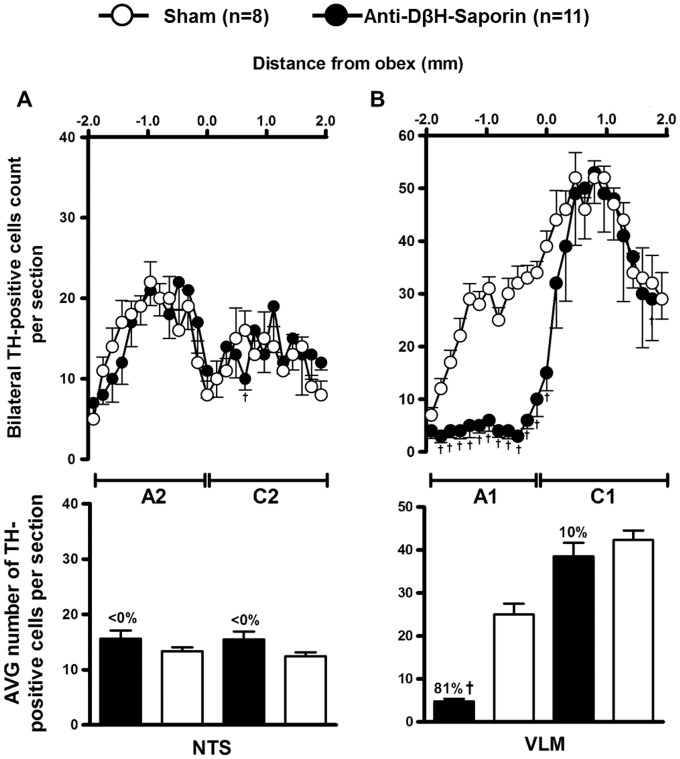
Lesions of A1 noradrenergic neurons after nanoinjections of anti-DβH-saporin into the CVLM. The number and average (mean ± S.E.M.) of TH-positive cells in 40-µm-thick sections of the dorsal (A) and ventrolateral medulla (B). Sections from animals that were submitted to A1 lesions or sham were taken from 1.9 mm rostral to the obex to 1.9 mm caudal to the obex. Bilateral nanoinjections of anti-DbH-saporin into the CVLM produced a loss of TH-containing neurons in this area (A1 group, loss  = 81%). However, in the RVLM (C1 group, loss  = 10%) and NTS (A2/C2 groups, loss < 0%), this loss was either decreased or not evident, respectively. ^†^ compared with sham, p<0.05.

TH-positive neurons are found within the ventrolateral medulla (VLM) and the nucleus of the solitary tract (NTS) from positions of approximately 1900 *μ*m caudal to 1900 *μ*m rostral to the obex (Figures 1 and 2). In saporin-treated animals (sham; n = 8), tyrosine hydroxylase (TH)-positive neurons in the VLM between 200 *μ*m and 1900 *μ*m caudal to the obex averaged approximately 25 cells per section (A1 neurons). In rats that were treated with bilateral nanoinjections of anti-DβH-saporin into their CVLM (n = 11), the number of TH-positive neurons that were caudal to the obex was reduced to approximately four cells per section. In this region, which encompasses the area of the A1 noradrenergic neurons, the number of TH-positive neurons was reduced by 81% when compared tosaporin-treated animals (Figures 1 and 2).

Nanoinjections of anti-D*β*H–saporin within the CVLM did not reduce the number of TH-immunopositive cells in the region that was located between 200 *μ*m caudal and 1900 *μ*m rostral to the obex (i.e., the region encompassing the C1 cell group; Figures 1 and 2). No significant changes in the number of TH-immunopositive cells (reduction of 0%; Figures 1 and 2) in the A2 or C2 cell groups of the NTS (between 1900 *μ*m caudal to 1900 *μ*m rostral to the obex) were seen.

**Figure 3:**
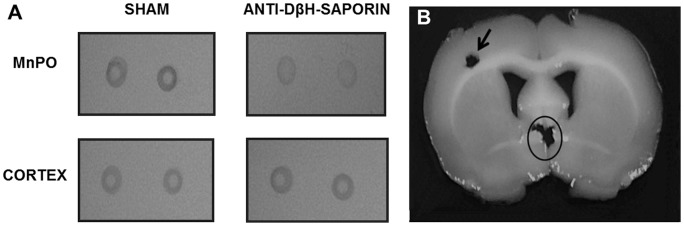
Photomicrography of the nitrocellulose membrane with marking of TH in the MnPO and cortex. Molecular analysis of noradrenergic neurotransmission of the A1 group of the CVLM region for MnPO with marking for TH (circle) by the Dot Blot technique in sham and anti-DβH-saporin-treated rats. Photomicrographs of a coronal section of a brain showing withdrawal of the tissue mass from the MnPO (circle) and cortex (arrows; B).

The Dot Blot technique, which was performed using samples of median preoptic nuclei (MnPO), showed that nanoinjections of anti-DβH-saporin in the CVLM reduced TH levels in the MnPO (Figure 3). This finding reveals that lesions in A1 were effective in altering the A1–MnPO noradrenergic pathway.

### Effects of A1lesions on the control ofthe baroreflex and chemoreflex

The baseline values of body weight (b.wt.), mean arterial pressure (MAP) and heart rate (HR) that were sampled before the infusion of HS are shown in Table 1. When compared with the control, animals that were nanoinjected with anti-DβH-saporin presented a basal tachycardia (HR: 396±13.4 vs. 343±8.4 bpm; P<0.05). Other values were similar to those of the sham and A1-lesioned rats.

**Table 1:** Baseline values for body weight, MAP and HR in sham and A1-lesioned rats.

Group	b.wt. (g)	MAP (mm Hg)	HR (bpm)
**Sham**	323±5.7	112±2.1	343±8.4
**A1 Lesion**	318±7.4	114±1.9	396±13.4^†^

Values are the means ± S.E. b.wt., body weight; MAP, mean arterial pressure; and HR, heart rate. ^†^ compared with the sham. p<0.05.

**Figure 4:**
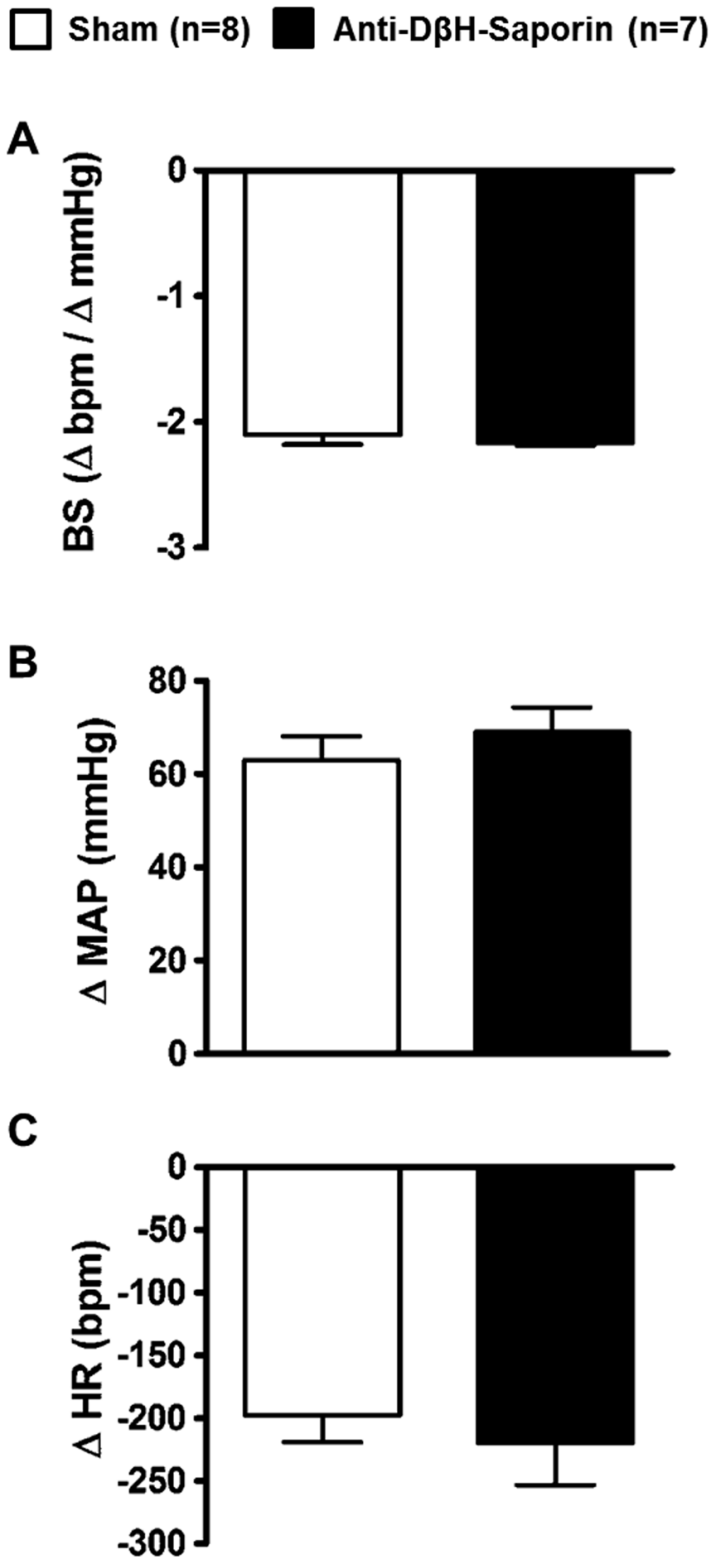
Baroreceptor sensitivity and chemoreflex response. Baroreflex sensitivity(BS; A) expressed by changes in the heart rate (HR) and mean arterial pressure (MAP) that were induced by phenyleprine infusion in sham and A1-lesioned rats. p<0.05. Changes in MAP (B) and HR (C) induced by intravenous potassium cyanide infusion.

The sensitivity of the baroreceptor reflexes, as evaluated by phenylephrine infusions, was not different between the sham (−2.1±0.07 bpm • mmHg^−1^; n = 8; Figure 4 A) and A1-lesioned (−2.1±0.02 bpm • mmHg^−1^; n = 7; Figure 4 A) groups.

Figures 4 B and C present the mean maximal changes in the MAP and HR that wererecorded during the chemoreflex test, and no differences inthe MAP (ΔMAP: 69±5.3 vs. 63±5.1 mmHg mmHg) and HR (ΔHR: −220±33.4 vs. −198±21.2 bpm) responses between A1-lesioned (n = 7) and sham (n = 8) rats, respectively, were evident.

### Effects of A1 lesions on the cardiovascular responses that are caused by hypertonic saline infusions

**Figure 5:**
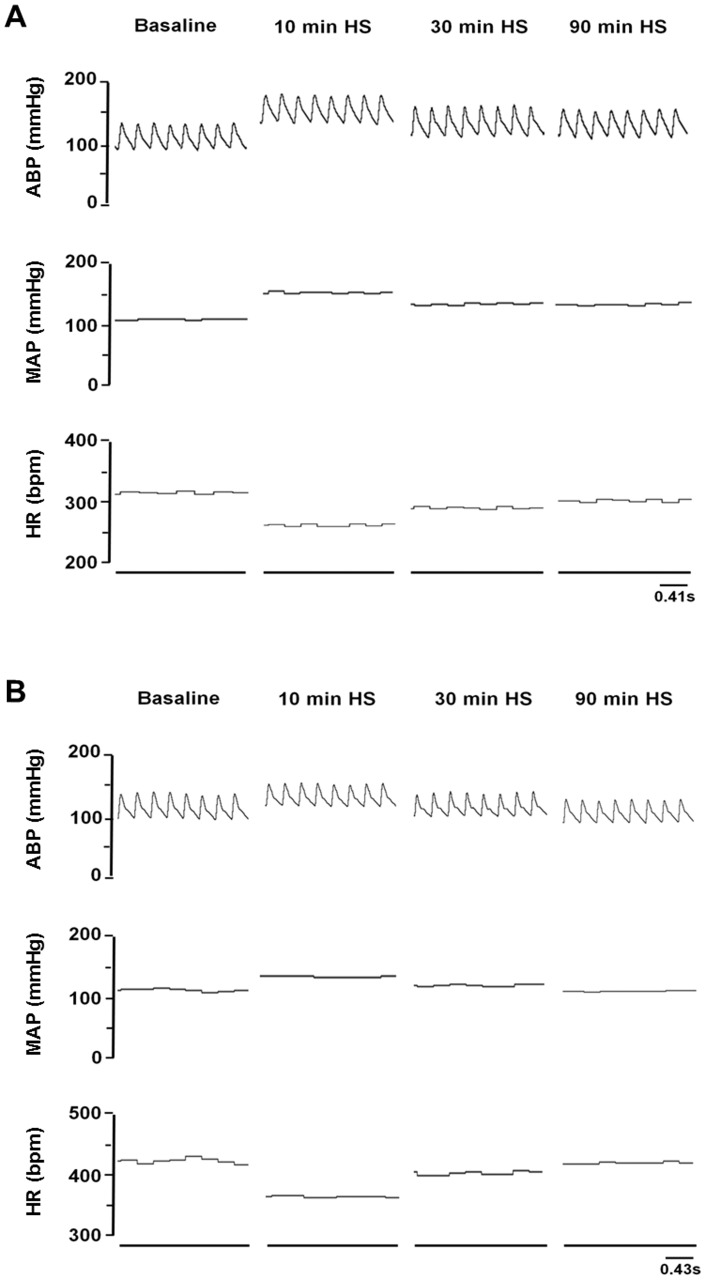
Typical examples. Digitized record of cardiovascular responses that were induced by hypertonic saline infusion in sham (A) and A1-lesioned rats (B). Arterial blood pressure (ABP), mean arterial pressure (MAP), and heart rate (HR).

**Figure 6:**
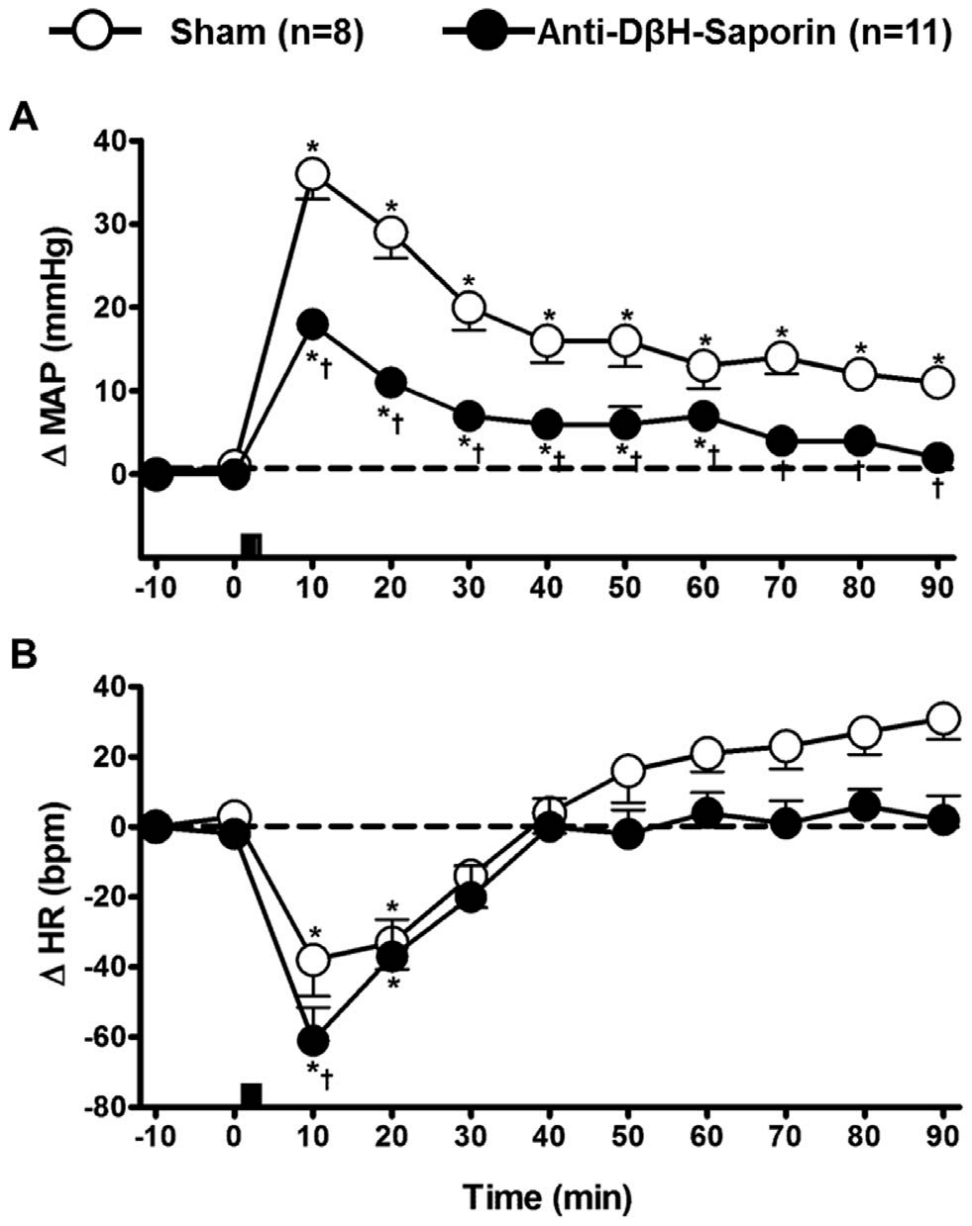
Effects of A1 noradrenergic neuron lesions on cardiovascular responses that were induced by hypernatremia. Effects from hypertonic saline infusion (3 M NaCl, 1.8 ml.Kg^−1^ body weight) on the mean arterial pressure (MAP; A) and heart rate (HR; B) of sham and A1-lesioned rats. The bars indicate the moment of hypertonic saline infusion. *compared with the baseline; ^†^ compared with the sham at the same time point;for both, p < 0.05.

In sham animals (n = 8; Figures 5 A and 6 A), HS infusions caused a sustained pressor response (ΔMAP: 36±3 and 11±1.8 mmHg, at 10 and 90 min, respectively; P<0.05 vs. baseline; n = 8). In anti-DβH-saporin-treated rats (n = 11; Figure 5 B and 6 A), pressor responses that were caused by HS infusionswere attenuated (18±1.4 and 2±1.6 mmHg, at 10 and 90 min after HS, respectively; P<0.05 vs. sham). In both groups, sodium overload triggered bradycardia, which was evident 10 min after HS (Figures 5 and 6 B; P<0.05 vs. baseline). However, the range of this HS-induced negative chronotropy was greater in the A1-lesioned group(ΔHR: −38±10.3 vs. −61±9.5 bpm; P<0.05) than in the sham group. In both groups,it was possible to observe heartbeat values that were similarto the baseline value approximately 30 min after HS infusion(Figure 5B).

### Effects of HS infusion on plasma sodium and blood hemoglobin concentrations

**Figure 7:**
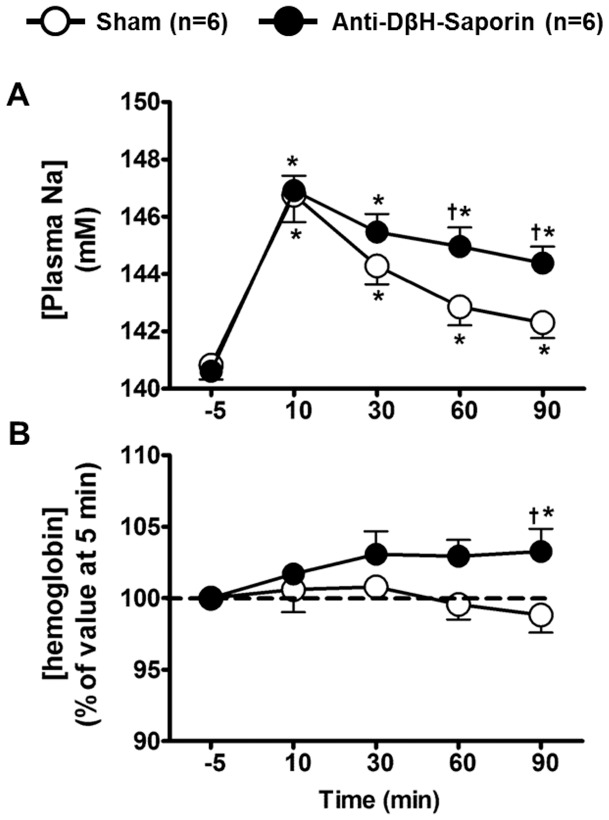
Sodium and blood hemoglobin concentrations in plasma. Mean ± S.E.M. of plasma sodium (A) and blood hemoglobin concentrations (B) at baseline and as a response to HS infusion in sham and A1-lesioned rats. * different from baseline; † different from sham at the same time point; for both, p<0.05.

Plasma sodium and hemoglobin outcomes were determined in sham and A1-lesioned rats before and after HS infusion. Baseline plasma sodium concentrations were similar in the sham (140.8±0.5 mM; n = 6; Figure 7 A) and A1-lesioned rats (140.6±0.3 mM; n = 6; Figure 7 A) 10 min after HS infusion, and plasma sodium levels increased similarly in both groups (146.7±0.9 mM and 146.9±0.5 mM in sham and A1-lesioned rats, respectively; Figure 7 A). Interestingly, the sodium concentration in anti-DβH-saporin-treated animals remained higher than that of the sham group throughout the experimental trial(Figure 7 A), and a significantincrease in the blood hemoglobin levels in A1-lesioned rats was observed 90 min after HS infusion (103.3±1.6% vs. 98.8±1.2%, A1-lesioned vs. sham rats, respectively; P<0.05; Figure 7 B).

### Effects of HS infusion on urinary excretion

**Figure 8:**
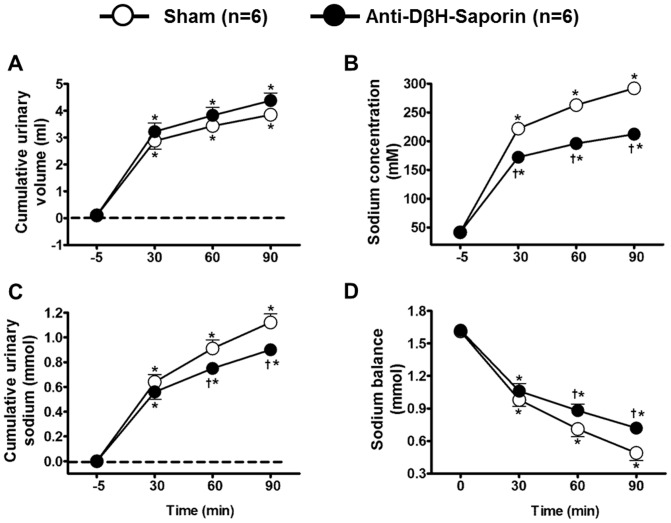
Effects of A1 noradrenergic neuron lesions on the renal responses induced by hypernatremia. Effects of hypertonic saline infusion (3 M NaCl, 1.8 ml.Kg^−1^ body weight) on the cumulative urinary volume (A), sodium concentration (B), cumulative urinary sodium (C) and sodium balance (D) of sham and A1-lesioned rats. Error bars indicate the S.E.M. *compared with the baseline; ^†^ compared with the sham at the same time point; for both, p<0.05.

Urine collectionduring the experiments showed that hypertonic saline increased the urinary volume of sham (n = 6) and anti-DβH-saporin-treated rats (n = 6; Figure 8 A), and this effect was enhanced 90 min after HS infusion (3.85±0.2 and 4.38±0.2 ml, sham and A1-lesioned, respectively; P<0.05 vs. baseline). A1-lesioned rats presented lower urinary sodium concentrations (212.09±7.1 mM, 90 min after HS infusion; Figure 8 B) and less cumulative urinary sodium (0.90±0.05 mmol, 90 min after HS infusion; Figure 8 C) than the sham group (292.0±6.8 mM, 90 min after HS infusion; 1.12±0.07 mmol, 90 min after HS infusion, respectively; Figures 8 B and C). While sham animals excreted 80% of their sodium, A1-lesioned rats excreted only 47% of their sodium after 90 min of HS infusion. Overall, these results indicate a lower capacity of A1-lesioned rats to excrete sodium.

## Discussion

Our most important findings demonstrated that lesionsin the A1 group caused the following: i)modification of the A1 – MnPO noradrenergic pathway; ii)an increase in the resting heart rate; iii) noeffect on baroreflexesor chemoreflexes; iv) an attenuated hypertensive response to hypertonic saline infusion; and v) a reduced ability to recover physiological osmolality by lowering sodium excretion.

Previous studies have indicated that A1 noradrenergic neurons are involved in the regulation of body fluid homeostasis [12], [17], [33]. Whether these neurons play a role in organizing cardiovascular and renal responsesthat are induced by changes in the volume or composition of the extracellular compartment is unclear. In this regard, our results pioneered the field in revealing that the A1 plays a pivotal role in recovering cardiovascular and renal homeostasis after hypertonic infusion. The present study provides the following new key observations: i) the pressor response to hypernatremia is attenuated in A1-lesioned rats; and ii) A1 noradrenergic neuron lesions slow the mechanisms of sodium excretion that are induced during acute hypernatremia in non-anesthetized rats. Thesefindings support the idea thatA1 neurons serve as a part of a neural pathway that is involved in the control of cardiovascular and renal responses that are induced by acute changes in the sodium concentration of the plasma. These renal outcomes exemplify the involvement of the A1 in the regulation of renal sodium excretion.

The present study demonstrates that nanoinjections of anti-D*β*H–saporin into the CVLM of rats produced an extensive depletion of the catecholaminergic cell population. Our data further show a massive lesion of the A1 catecholaminergic cell group because bilateral nanoinjections of anti-D*β*H–saporin induced a marked loss (81%) of TH-containing neurons(within 1.9 mm to 0.2 mm caudal to the obex). In addition, no significant changes in the catecholaminergic neurons of the rostral ventrolateral medulla(RVLM; C1 group; 10%) and NTS (A2 group; <0%) regions were seen, which indicates that the adopted method was effective and specific to damaged A1 neurons.

Previous reports support the assertion that a loss in immunoreactivity is indicative of neuronal death [17], [49], [50]. The mechanism of specific internalization of anti-D*β*H–saporin implies that only cells synthesizing D*β*H can take up the conjugated toxin. It has been postulated that the somatodendritic exocytosis of catecholaminesin catecholaminergic neurons resulted in the exposure of DβH and, therefore, the possible internalization of the anti-DβH-saporin complex via their soma and/or dendrites. It is not likely that the noradrenergic neurons survived because saporin acts by blocking the ribosomal 60S subunit to inhibit protein synthesis.

Wrenn et al. [51] reported a lack of TH or DβH-cells 9 months after intracerebroventricular injections of anti-DβH–saporin, thus demonstrating the irreversible effects of this substance. Similar results were obtained in a recent study in which nanoinjection of anti-DβH–saporin into the CVLM depleted approximately 79% of A1 noradrenergic neurons within this region [17]. Furthermore, in the present study, the A1 neuron lesions revealed a decrease in the levels of the TH enzyme in MnPO, which suggeststhat damage in the CVLM to MnPO noradrenergic pathway occurred. These results indicate the efficacy of anti-DβH-saporin in the selective destruction of noradrenergic neurons.

Studies have demonstrated that the noradrenergic A1 neurons that receive inputs from baroreceptors and cardiopulmonary receptors [38]–[40] project to the diencephalic structures that are involved in regulating thecirculating volume. For example, neuroanatomical studies provided consistent evidencethatMnPO and paraventricular hypothalamus(PVN)receiveddense noradrenergic inputs [41]–[48]. Nearly 80% of the A1 noradrenergic neurons of the CVLM region project directly to the PVN and MnPO [48]. Accordingly, functional *in vivo* electrophysiology assessments have demonstrated that these noradrenergic projections from the A1 to the hypothalamic regions are mostly excitatory [46], [47], [52]–[56]. Using extracellular recordings of PVN neurons, Saphier [46] demonstrated that electrical stimulation ofthe A1 region excited most of the magnocellular oxytocin- and vasopressin-excreting PVN neurons. Similarly, Tanaka *et* *al.* [56] described that electrical stimulation of the A1region promoted excitation of MnPO neurons that projected to the PVN viathe alpha-1 and alpha-2 adrenoreceptors. This evidencestrongly suggests an excitatory noradrenergic projection from A1 to PVN and MnPO, which are mediated by alpha-adrenergic receptors.

Empirical evidencehasshown the involvement of A1 and MnPO in cardiovascular regulation, and current data showed that A1 lesions and a decrease in TH levels in the MnPO did not change the baseline levels of mean arterial pressure. These results are in full agreement with those of Pedrino et al. [12], [17], who also reported unaltered baseline values of MAP between sham and A1-lesioned rats. Moreover, in the present study, the baseline tachycardiathat wasfound in A1-lesioned rats indicatedthe contribution of this medullary areato the control of the resting HR. Previous studies did not report this baseline tachycardia [12], [17]; however, in those studies, the animals were anesthetized. We hypothesize that A1 neuronsmay directly or indirectly influence hypothalamic pathways that govern the baseline heart rate. As shown in this report, it is not unlike to suggest that these pathways may be the same responsible for slowing sodium excretion to HS infusion. This idea seems plausible because osmoreceptorsmodulate hypothalamic regions activitythat control renal and cardiac sympathetic supplies, such as the PVNand MnPO [6].

The baroreflex and chemoreflex are important mechanisms that are used to recover homeostasis after changes in extracellular volume and composition occur [10], [26], [57]. Previous studies demonstrated that the hypertension that was induced by HS was significantly higher in sinoaortic,denervated rats [10]. Moreover, the bradycardia, which was induced by HS infusion,wasabolished, indicating the involvement of the baroreflex in this response [58]. In spite of the differential MAP and HR responses to HS infusion in A1-lesionedanimals, we found a preservation of the baroreflex and chemoreflex, which suggests that other mechanisms, possibly supramedullary,underliethe differences in the pressor and bradycardic responses in A1-lesioned rats that were infused with HS. However, this idea must be further investigated.

Consistent with previous results using the same protocol [28], in the present study,HS infusion induced an increase in plasma sodium levels without changing the blood hemoglobin concentration in sham animals, and this treatment resulted in a slight increase in A1-lesioned rats. Therefore, it is conceivable that cardiovascular adjustments that are induced by HS infusion, as described in the present study, are due to the increased sodium concentration rather than secondary to volume expansion.

Many efforts have been attempted to understand the mechanisms that are involved in the cardiovascular responses to HS infusion [10], [15], [17], [18]. Similar to the resultsthat were obtained in the present study, HS infusion caused hypertension in intact rats [10], [16], [49], [59], [60]. The pressor responses appeared to be due to an increase in lumbar sympathetic nerve activity and vasopressin release [10], [59], [60]. In this study, we observed that A1 lesions attenuated the increase in blood pressure that was caused by HS. We suppose thatA1-lesioned rats present a lower capacity to release vasopressin. As mentioned before, Saphier [46] demonstrated that electrical stimulation of the A1 region excited most of the magnocellular neurons that excreted oxytocin and vasopressin. Consistent with these findings, Kapoor&Sladek [61] demonstrated that the alpha(1)-adrenergic agonist phenylephrine increasedvasopressin release in the hypothalamoneurohypophyseal axis. Therefore, these results suggest that neuronal projections from the A1 to paraventricular and supraoptic nuclei may be involved in vasopressin release in response to acute hypernatremia.

Reduced fractional sodium excretion indicates that A1 lesions impair the capacity of the augmenting glomerular filtration rate, which is a possible renal mechanism determining the increases in sodium loss in response to hypertonic NaCl. Several factors may have contributed to the attenuation of this effect in A1-lesioned rats. The small pressor responsemight prevent the low natriuresis that was observed in A1-lesioned rats that had been infused with HS. This response could also attenuate renal sympathoinhibition because renal denervation prevents increases in renal sodium excretion evoked by hypertonic NaCl [19]. We have provided substantial evidencethat the renal vasodilation and sympathoinhibition that was induced by HS infusion were blunted in animals that were submitted to A1 noradrenergic neuron lesions [12], [17]. In addition, Frithiof et al., [62] have demonstrated that intracerebroventricular administration ofhypertonic NaCl decreasedthe sympathetic outflow to renal nerves in conscious sheep. Our main hypothesis states thata reduction in the hypertensive response that is associated with an attenuation of sympathoinhibition would reduce the sodium excretion that is induced by hypernatremia in A1-lesioned rats. To the best of our knowledge, this is the first statement on the role of A1 noradrenergic lesions in hypertension and renal sodium excretion responses that are induced by hypernatremia in non-anesthetized rats.

We conclude that the recruitment of A1 noradrenergic neurons is essential toadjustthe cardiovascular and renal responses that are induced by acute changes in the concentration of plasma sodium. These noradrenergic neurons are integral parts of pathways that are involved in the response to hypernatremia because the lack of these cells results in inefficient responses. Dysfunctions in this system could contribute to the genesis and aggravation of cardiovascular and renal diseases that are likely related to renal sodium retention such as hypertension, cirrhosis and cardiac failure.

## Methods

### Animals

All experiments were conducted on adult male Wistar rats (280–340 g), which were obtained from the central animal house of the Federal University of Goiás. Experimental procedures were designed with strict adherence to the National Institute of HealthGuidelines for the Care and Use of Laboratory Animals as approved by the Ethics Committee, Federal University of Goiás (protocol number 172/09).

### Nanoinjections of anti-DβH-saporin or saporin into the CVLM

Animals were anaesthetized with halothane (2%; Cristália Ltda, Itapira, SP, Brazil) in O_2_ and mounted prone in a stereotaxic apparatus (David Kopf Instruments, Tujunga, CA, USA) that had its incisor bar 11 mm below the interaural line. After partial removal of the occipital bone, the meninges covering the dorsal surface of the brainstem were cut and retracted, and the *calamus scriptorius* was visualized. Nanoinjections of anti-DβH-saporin (0.105 ng.nL^−1^; Advanced Targeting Systems, San Diego, CA, USA) or an equimolar dose of saporin (0.021 ng.nL^−1^; Advanced Targeting Systems, San Diego, CA, USA) were made bilaterally at two levels of the CVLM. For all nanoinjections into the CVLM, a glass micropipette was positioned as follows: 0.3 mm rostral and 0.3 mm caudal from the *calamus scriptorius*, 2.0 mm lateral from the mid-line, and 2.0 mm ventral from the dorsal surface. These co-ordinates were based on the region of the CVLM consisting of the A1 group [48]. After nanoinjections, the micropipette was left in place for 3 min, the incision wasclosed, and the animals were placed on a heated pad to maintain their body temperature during recovery. A prophylactic antibiotic dose (penicillin, 60.000 IU • kg^−1^, i.m. Sigma-Aldrich, St Louis, MO, USA) was injected after surgery, and the animals were studied 15 days after microinjections into the CVLM.

### Surgical procedures

The rats were anesthetized with 2% halothane (Cristália Ltda, Itapira, SP, Brazil) in O_2_, andthe right femoral artery of each rat was cannulated for blood pressure recording. The right femoral vein was catheterized for drug administration (phenylephrine and potassium cyanide) and infusion of hypertonic saline (HS), and the cannulas were led subcutaneously to exit between the scapulae.

### Recording of arterial pressure and heart rate

To record the arterial pressure of each rat, an arterial catheter was connected to a pressure transducer that was attached to a bridge amplifier (ETH-200; CB Sciences, Dover, NH, USA). The pulsatile pressure of each rat was recorded continuously with a PowerLab System (ADInstruments, Colorado Springs, CO, USA), and the MAP and HR were determined using the pulsatile signal and Chart software (version 5.5.6; ADInstruments, Colorado Springs, CO, USA).

### Baroreflex and Chemoreflex Tests

A day after surgery, the animals were subjected to baroreflex and chemoreflex tests through the infusion of phenylephrine (1 μg, 1.5 μg and 2 μg; adrenergic agonist; Sigma-Aldrich, St Louis, MO, USA) and potassium cyanide (40 μg; Sigma-Aldrich, St Louis, MO, USA), respectively. All injections were performed with at least 5 min between each injection or until cardiovascular parameters returned to baseline.

### Infusion of hypertonic saline and blood sampling

To increase the concentration of sodium in the plasma of the rats, HS (3 M NaCl, 1.8 ml • kg, b.wt., over 1 min) was infused through the femoral vein cannulas. After infusion with HS, the cardiovascular parameters were recorded for 90 min, and blood samples (0.20 ml each) were withdrawn from the arterial cannula 5 min before and 10, 30, 60 and 90 min after HS infusion. After each sample was collected, an equal volume of sterile 0.15 M NaCl was injected to reduce the changes in the extracellular fluid volume due to sampling. The blood hemoglobin concentration was measured immediately using a kit from Sigma (Drabkin's reagent, kit 525; Sigma-Aldrich, St Louis, MO, USA), and the remainder of the sample was centrifuged for 5 min at 6000 g. The plasma was removed and stored at −20°C, and the sodium concentration of the plasma in the blood was measured using a flame photometer (model DM6, Digimed, São Paulo, SP, Brazil).

### Urine Analysis

The A1-lesioned and sham rats were housed in metabolic cages with free access towater for only 48 h before tests were performed. On the day of the urinary excretion test, all animals received a suprapubic massage to induce micturition. After 5 min, the HS solution was infused, and urine samples were collected in polypropylene tubes to measure the urinary volume at 30, 60 and 90 min. The concentration of sodium in the urine was measured using a flame photometer (model DM6, Digimed, São Paulo, Brazil), and the amount of sodium in the urine was determined by calculating the product of the urine volume and sodium concentration. The urinary balance was calculated as the difference between the amounts that were infused and excreted. During the experiments, the animals had no access to water or food.

### Perfusion, fixation and tissue collection

At the end of the experiments, the animals were profoundly anesthetized and perfused through the heart with saline (0.15 M NaCl) followed by a solution of 4% paraformaldehyde (Sigma-Aldrich, St. Louis, MO, USA) in 0.2 M sodium phosphate buffer (500 ml at pH 7.4).The brain of each rat was removed and post-fixed in 4% paraformaldehyde solution for 1–2 h and then cryoprotected in 30% sucrose solution. Coronal sections of 40 *μ*m of the brainstem were collected (in 4 serially adjacent sets) and stored in 0.02 M potassium phosphate-buffered saline (KPBS, Sigma; pH 7.4).

### Immunohistochemistry

Each fourth brainstem section was processed for immunohistochemical detection of TH. The sections were pre-incubated for 15 min in 3% hydrogen peroxide in 0.02 M KPBS followed by a 30-min incubation in 2% normal horse serum (Vector Laboratories Inc., Burlingame, CA, USA) in 0.02 M KPBS. The sections were incubated overnight at 4°C with mouse monoclonal antibody (1∶2000 dilution, ImmunoStar, Inc., Hudson, WI, USA) in 2% normal horse serum and Triton X 10% followed by a 1-h incubation with biotinylated horse anti-mouse IgG (Vector Laboratories Inc., Burlingame, CA, USA; 1∶2000 dilution with Triton X 10%). After these incubations, the sections were processed using the avidin–biotin procedure, which utilized Elite Vectastain reagents (Vector Laboratories Inc., Burlingame, CA, USA). Diaminobenzidine (DAB; Vector Laboratories Inc., Burlingame, CA, USA) was used to produce a brown, cytoplasmic TH reaction product. Sections were mounted on slides, dehydrated in a series of alcohols, cleared in xylene and coverslipped.

### Cell counting

Counts of neurons were performed in every fourth medulla oblongata section (40 of each 160 *μ*m). All immunolabeled neurons in the ventrolateral medulla (VLM; A1/C1) and nucleus of the solitary tract (NTS; A2/C2) were counted bilaterally to quantify the extent of the anti-D*β*H–saporin-induced lesion. The neurons were counted at x200 magnification using a Nikon light microscope (Eclipse Ni-U; Nikon Corporation, Tokyo, Japan).

### Dot Blot

Approximately 100 mg of MnPO and cortex brain samples were macerated in Tris-HCl (50 mM, pH 7.5), andthe samples were centrifuged at 12.000 rpmsand 4°C for 5 min. The supernatant was transferred to another tube, and the protein concentration was estimated using the Bradford method [63]. Fifty micrograms of each sample was transferred to a nitrocellulose membrane, andafter a 1-h blocking step in 5% powdered milk that was diluted in TBS 0.05% with Tween 20, the membrane was incubated overnight at 4°C with a mouse monoclonal TH antibody (1∶5000 in 1% milk TBS-Tween 20; Immuno Star Inc., Hudson, WI, USA). Following 4 washes for 15 min at RT, the membrane was incubated for 1 h with an anti-mouse antibody that was coupled to phosphatase (1∶2000 in 1% milk TBS-Tween20), washed again 4× and finally revealed using the BCIP/NBT solution (Amresco LLC, Solon, OH, USA).

### Data analysis

The effects of treating with anti-DβH–saporin or saporin on the number of catecholaminergic medullary neurons are presented as the means ± S.E., and cell countings for every section were compared by one-way ANOVA. Additionally, total cell counting for the A1, C1, A2 and C2 regions were calculated and compared between groups using the unpaired Students t-test. Data on cardiovascular and renal responses and plasma sodium and blood hemoglobin concentrations were analyzed by two-way analysis of variance followed by the Newman-Keuls test. The differences in the baseline levels (Table 1) between the groups were analyzed using the unpaired Student's t-test, and a value of P<0.05 was considered to denote a significant difference.
